# The Atlanta Society of Medicine

**Published:** 1892-03

**Authors:** 


### Society Reports.

### THE ATLANTA SOCIETY OF MEDICINE.

### MEETING OF JANUARY 19, 1892.

PRESIDENT, DR. KENDRICK, in the chair. Although an inclement night, the following gentlemen were present: Drs. Kendrick, Hardon, Kime, McEvoy, McRae, H. F. Harris, Duncan, Cooper, Hutchins and C. D. Roy.

The paper of the evening was read by Dr. Hutchins, subject, RINGWORM. See page 20.

*Discussion.*—Dr. Duncan had seen ringworm for many years. Chiefly in order to get rid of the cases, advised patients to burn common newspaper in a plate and sprinkle the residue upon the lesions. Has seen this effective.

DR. HARDON had treated cases in his early practice successfully. Gave history of the case of a member of the *demi-monde* who attributed a patch of the disease on her shoulder to a man's chin having rested there, he having ringworm of the beard.

DR. MCRAE had seen many cases. Found those of the scalp most tedious in treatment. Best results from the pyrogallic acid and collodion.

DR. MCEVOY had seen a few cases. Used with success bichloride of mercury, four grains, acetic acid, one ounce.

Closing the discussion, Dr. Hutchins said that, owing to the brittleness of the hairs and the tediousness of the operation, he had avoided epilation, and believed that the great capacity of penetration shown by the kerosene oil would obviate the necessity for epilation. Must destroy every vestige of the lesions before ceasing treatment.

C. D. ROY, M. D.,
Secretary.

### MEETING OF FEBRUARY 2, 1892.

The Society was called to order by the President, DR. KENDRICK. The following members were present: Drs. Cooper, Hardon, McRae, Richardson, Duncan, H. F. Harris, Grandy, Glass, Hutchins, Van Goidtsnoven, McEvoy, Kendrick and C. D. Roy.

The discussion for the evening was

### INFLUENZA; MANIFESTATIONS OF THE PRESENT EPIDEMIC.

Discussion was opened by DR. COOPER. He said: "In my experience the present epidemic of "grip" is much milder than the preceding. For convenience I have recognized the following types:

1. *Bronchial.*
2. *Naso-pharyngeal.*
3. *Gastric.*
4. *Rheumatic.*

The most frequent type was that with laryngeal (?) symptoms. The cases presented the usual symptoms of pain, aching in the limbs, flushing of the face, etc. The bronchial type was sometimes very severe, almost simulating pneumonia. The cough was harassing and prone to persist after the other symptoms had disappeared. Has had two or three cases of severe nasal congestion with its symptoms, followed by marked relief upon the discharge of a large amount of secretion from the nasal and accessory cavities. The attack of "grip" has usually left the patients in a very depressed state.

*Treatment.*—The Doctor does not believe in abortive treatment or specifics. "Make your patient comfortable; let the disease run its course, and build up with tonics in the declining stage. If the patient has fever he is rendered comfortable by reducing the temperature." The following pill has given him the best results:

℞. Phenacetin, gr. v.

Pulv. opii, gr. ss.

Camphor, gr. $\frac{1}{28}$.

Pulv. ipecac, gr. $\frac{1}{50}$.

In the cases of nasal stenosis he had used the following with almost magical effect: One pint of boiling water, a teaspoonful of compound tincture of benzoin, five grains of iodine crystals. The medicated steam thus formed is inhaled through the nose and mouth by the patient.

In the rheumatic type the salicylates have given the best results.

DR. DUNCAN had seen the "grip" in all its types. Had one or two cases with marked congestion of the kidneys. In the "gastric" type he has used with success two drops of dilute hydrocyanic acid with lacto-peptine. Agrees very closely with Dr. Cooper as to the general line to be pursued.

DR. GRANDY had used compound tincture of benzoin and turpentine in hot water, as inhalation, with success.

DR. GLASS aims at making the patient comfortable, which is best accomplished by keeping the temperature normal.

DR. MCRAE had met a type, in his practice, which he denominated the *afebrile*. This, as its name indicates, possessed all the symptoms without the rise in temperature. The succeeding prostration is very marked. He treats his cases "symptomatically."

DR. ROY said his cases had been more the *sequelæ* than the disease in its acuteness. Such sequelæ have been persistent bronchial cough, hoarseness and naso-pharyngeal catarrh. The hoarseness seemed to be a partial paresis of the laryngeal muscles similar to that in diphtheria, and hence showing its probable specific character. Used the ordinary methods of treating the sequelæ.

THE PRESIDENT said he was much pleased with the discussion. He had no complication with pneumonia in his cases, nor had he given quinine in the attacks. He prefers the coal tar antipyretics.

C. D. ROY, M. D.,
Secretary.

---

MEETING OF FEBRUARY 16, 1892.

Called to order by the President, DR. KENDRICK. Following gentlemen were present: Drs. Cooper, Crow, Duncan, Elkin, Gilbert, Hardon, N. O. Harris, H. F. Harris, Hutchins, Kendrick, Kime, F. W. McRae, F. B. McRae and McEvoy.

"Reports of Cases" was the order for the evening.

DR. CROW exhibited two specimens of ovaries removed. First, case of a negro woman, twenty-six years old, severe dysmenorrhœa. Patient reduced from loss of blood, and pain. Removed one ovary, which was covered with cysts from size of pea to that of an English walnut. The other, not removed, was small and almost cirrhotic. Patient made a good recovery—up in fourteen days. Had since had menstrual efforts, no blood, simply watery discharge. Second specimen, from patient aged twenty-nine. Childbirth, August, 1891, bad recovery. Dr. Crow first saw her a week before Christmas. Had whitish, purulent discharge from vagina. Subject to swelling and tenderness of lower part of abdomen; peritonitis and fever when first seen. Examination revealed piece of adherent placenta. Curetted uterine cavity and packed with iodoform gauze. The doctor thought he could make out evidence of pyosalpinx, and there were symptoms of recurrent peritonitis. Removed ovary two weeks ago. Found it containing a large suppurating cyst. Adhesions made the operation very difficult. Had to break them up following broad ligament by sense of touch and "peeling out" the ovary. It was the size of an orange and contained about half a pint of offensive, purulent fluid. Was not able to tie the vessels, but douched cavity thoroughly with hot water, using about ten gallons. Temperature did not rise above 100°, until eighth day, when there was a rise to 103°, due to hysterical attack. There was union of abdominal incision by first intention.

DR. HARDON asked if drainage was not used. Dr. Crow replied, "for only eighteen hours," thinking the longer use of the tube tended to cause formation of fistula. Preferred the removal of the tube after six hours. Patient claims to have passed a renal calculus to-day. Thought the abscess of ovary resulted from sepsis due to retained piece of placenta. The other ovary seemed normal, but was bound down by adhesions. Patient was weak and anæmic when operated upon. Had been in bed five months.

DR. HARDON said circumstances must govern the time of leaving in the drainage tube. His rule was to let it remain in as long as there was discharge of blood, and to remove it as soon as only serum was discharged through the tube. He inserts a suture at the time of putting in the tube, so as to be ready to close the opening immediately upon its removal.

THE PRESIDENT said that he had a patient in whom he felt a great deal of interest, whom he advised to have her ovaries removed by a specialist. She menstruated regularly after the operation.

DR. HARDON asked if the ovaries *were* removed. The President replied that the right one was, and the operator afterwards told him the left was "fatty degenerated."

DR. DUNCAN reported some cases of "eruptive trouble" in children who are sick only a few hours, eruption then occurring. Eruption chiefly macular, somewhat resembling measles, although most of the patients had had measles. Diagnosed "roseola" or "erythema." Has had some doubt as to how to classify the cases and what kind of a certificate to write where they were school children. Doubtful as to contagion. There was some enlargement of anterior and posterior cervical glands. Treated the cases with mild aperients, tinct. rhus. and phenacetin.

THE PRESIDENT had seen six or eight c[illegible] hese cases. Called it roseola. Enlargement of posterior cervical glands. Well in a day or two.

DR. DUNCAN thought the disease probably contagious.

DR. MCRAE had seen several cases, only one in a household. Did not consider it contagious.

DR. COOPER had seen cases. There was slight fever, suffusion of eyes, a little eruption on face, more on neck, profuse on body. Submaxillary glands enlarged. Called it "German measles."

DR. CROW said Coe, of New York, called it a glandular fever. Seemed somewhat epidemic. Treatment—*expectant*.

DR. ELKIN had seen two cases in one family, another solitary. Cervical glands enlarged. Diagnosed roseola.

DR. HARDON reported a case of ovarian cyst in a patient fifty-eight years old. Developed within a year. Abdomen as large as of a seven months pregnancy. Patient not very uncomfortable. Operated the preceding Wednesday. Evacuated a fluid resembling bile. Tumor universally adherent over anterior surface. Not adherent posteriorly. After evacuation of fluid found a secondary cyst in posterior superior part one-fourth size of the whole tumor, filled with white, very thick colloid matter. Removed, necessarily, without evacuation. Flushed and sponged abdominal cavity. Put in his (usual—rubber) drainage tube. Temperature did not exceed $100\frac{2}{5}°$. Patient could not evacuate the bladder for eight days after the operation. Attributes this to temporary distention paralysis of abdominal wall. Estimated the weight of tumor at thirty pounds.

DR. ELKIN showed specimens from a case of abscess of left kidney. Specimens removed after death. Woman of twenty-three or twenty-four years. Health "failed" eighteen months ago. (Tuberculosis in the family.) In a few months swelling developed in left side, over the kidney, leading to suspicion of abscess. Aspiration disclosed pus. Cut down along outer border of *quadratus lumborum* muscle. Could feel a cavity as large as a pigeon's egg. Pus seemed to have burrowed beneath the capsule. Flushed with bichloride 1–2000 and put in a thick rubber tube, through which urine was discharged. Patient did very well for ten days. Then rise of temperature; loss of appetite and flesh. Enlarged the incision and found the pus had dissected towards crest of ilium and to region of tenth rib. Thinks this was because of tube being, perhaps, too small. Inserted into purulent "pockets" three tubes. Patient very well for ten days, then got worse; died. Examination for tubercle bacilli, by Dr. H. F. Harris, negative. Frequent urination was a characteristic until after the operation.

DR. CROW had had a similar case. Called on account of rigor. Tempearture 103°. Enlarged kidneys, pus upon aspiration. Cut down into kidney next day. Washed out with bichloride solution, then hydrogen peroxide diluted one-half, for two weeks, until irritation was produced, then used listerine. Full recovery. Finds seventy-two recorded cases of recovery. His makes seventy-three. Thought trouble in Dr. Elkin's case was due to too small a drainage tube. Wound still open in Dr. Crow's case a year after operation.

DR. COOPER said the nearest like Dr. Elkin's case which he had had was one of perinephritic abscess. Patient emaciated, weighing not over ninety pounds. There was no tumor. Diagnosis by presence of irritation of *psoas magnus* producing flexion on thigh. Aspirating needle found pus, the second time aspirated. Recovered after operation.

Also reported and showed specimen from a case of great rarity. It was a tumor (solid) from right ischio-rectal fossa of a lady aged thirty-five. About the size of a hen egg. Removed *en masse* leaving a large cavity which was closed by sutures as for repair of ruptured perineum, thus getting complete union by first intention. The tumor was opened in the presence of the Society. The doctor thought diagnosis lay between sebaceous cyst and fatty tumor. No fluid had come through aspirating needle. The tumor being opened it was found to be a *sebaceous cyst.*

Reported, also, urethrotomy in man of forty-two years. Stricture for seven or eight years. Patient weak, anæmic, had hemorrhages from lungs and cystitis. Operation and dilatation some years before, but stricture returned, owing to neglect of using bougie. A homœopathic had promised a cure by medicine internally, and *vis medicatrix naturæ!* Urine contained much mucus and was loaded with albumen. Stricture so tight that the introduction of a filliform was made with difficulty, after a month's effort. During etherization an old cavity in lung ruptured and a great amount of pus discharged from mouth. Did the "combined" operation successfully. Stricture almost cartilaginous. Put full sized catheter in opening. Left for eight and one-half days.

DR. McRAE, referring to the case of sebaceous cyst, mentioned one occurring beneath the tongue, which he had reported to the Society.

He said he did seven perineal sections for stricture during 1891. Thinks he was the first in Atlanta to employ perineal drainage. Mentioned case of severe, impassable, cicatricial stricture upon which he operated *without a guide.* Found the drainage tube very useful. Retains with T. bandage. Results were satisfactory. Attached piece of rubber tubing to drainage tube to conduct the urine into a vessel by the bedside.

DR. MCRAE also reported a case of convulsions in pregnant woman, relieved by podalic version. First convulsion six weeks before operation. Diet; chloroform and veratrum internally. Upon next attack chloroformed and delivered as above. Os contracted in mouth of child; impossible to relax for fifteen minutes; asphyxiation. Mother made a good recovery.

DR. MCEVOY: "On the 25th day of January, 1892, I was called to see a negro man thirty years of age, of fairly good general condition. The history of his case was as follows: About five years previous to my visit, while engaged in lifting some heavy weight, he suddenly felt something give way in the lower portion of his bowels. Upon investigation he discovered a small tumor in the right inguinal region; this was accompanied with but slight pain. Upon going to his bed and lying on his back it disappeared, only to reappear again upon the slighest provocation. This state of affairs had been going on for the past five years, but each time the tumor appeared it could easily be made to disappear by the method used by the patient when first noticed until about ten days before my visit. Upon examination I not ced considerable swelling of the scrotum, pyriform in shape. The longest diameter of the tumor was about eight inches. The scrotal walls seemed to be quite thickened, and fluctuation could not be detected. The patient's pulse was 110, temperature 98°. Bowels costive, slight headache, considerable nausea and some vomiting. The tongue was covered with a brown coating. Although the history of the case given by the patient was identically that of hernia, as were also the present symptoms, yet there were evidently also manifestations of a hydrocele of the tunica vaginalis testis.

DR. J. McF. GASTON was invited to see the case with me, and after an examination concluded that while the tumor had all the appearance of a hydrocele, yet from the history of the case as was obtained from the patient, he thought a hernia might also be present but slight taxis proved to be of no avail. The patient was anæsthized and again reduction was attemped, both by Dr. Gaston and myself, but without success, until it was concluded that any further interference in this manner would prove harmful. A hypodermic needle was then introduced into the lower portion of the tumor and the piston filled with a clear reddish fluid was withdrawn. This was to our minds conclusive evidence. In connection with other features in the case that whatever other trouble might exist, we had evidently to deal with a hydrocele. The patient's bowels being costive, a prescription was given as follows :

℞. Hydrarg. chlor. mitis.,
Soda bicarb., aa. gr. v.
M. Ft. chart. no. j.

Sig.—To be taken at once, followed by a good dose of oil should the bowels fail to act in seven or eight hours.

Upon returning the following day, I found the patient's condition about the same as the day previous, with the exception of a slight acceleration of temperature with also a slight pain in the abdomen. The patient had vomited about twice during the night. From the evidence of an accumulation of fluid in the sac, as was proven by the hypodermic syringe the day previous, I concluded that I would withdraw the fluid and treat the hydrocele by the method "advised by Livis and recommended by Dr. John A. Wyeth," as follows: The tumor was shaven in its anterior aspect and cleansed thoroughly. About fifteen drops of a four per cent. solution of cocaine was injected in such a way that local anæsthesia was obtained through the walls throughout an area of half an inch in diameter. A trocar and canula was then introduced through this anæsthetized zone at a point about one-third the distance from the lower portion to the upper along the anterior aspect. The stylet was withdrawn and about one-half pint of fluid escaped. The tumor was reduced to about one-fourth its former size. Twenty drops of pure carbolic acid was then injected into the sac through the canula and the scrotum pressed so as to distribute the acid over the entire surface. The operation was followed with very little pain and the patient expressed himself as being considerably relieved.

The following day the tumor began to swell, which continued for about a week as though it was refilling, but on the seventh day it began to subside. This decreased gradually until the tenth day. The ninth day, however, I was called in haste to see the patient and found him suffering intensely with pain in the abdomen. Bowels still costive, some vomiting, etc. Upon examination, I discovered a considerable swelling in the right inguinal region very tender to the touch and œdematous. The patient was given an enema which caused a slight action, and then placed on the following prescription to be taken in teaspoonful doses every two hours.

℞. Antimonii et potass. tart., gr. ss.

Tr. opii, ℥i.

M. Aquæ camphoræ, q. s. ad. ℥iv.

Hot applications were also used to the abdomen. This had the effect of reducing the pains to a considerable extent, also reducing the nervous irritability, which was extreme, in the case.

The tenth day the symptoms were more favorable. The vomiting and pain were less severe and the patient seemed to be resting comparatively easy. On the eleventh day, however, his temperature was 104½°, pulse weak, severe pains over the abdomen and tympanites. The patient died from peritonitis the following night.

This case, gentlemen, is of considerable interest to me, because it is very seldom that we find a hydrocele of the tunica vaginalis testis communicating with the peritoneal cavity as was evident in this case, and while the patient's condition from the beginning pointed to a hernia, yet there was certainly no strangulation as was shown by the fact that the patient's bowels could be influenced by cathartics, and also the absence of food and stercoraceous matter in the matter which was vomited. The patient's death "in my opinion" was due to an extension of inflammation from the sac to the peritoneal cavity, thereby producing a fatal peritonitis."

DR. MCRAE said he was very much interested in the case, but wished to criticize—in a kindly spirit. Thought it would have been better to have incised the sac, stitched edges to borders of wound, packed the sac and thus given proper treatment for hydrocele, and time to clear up the diagnosis.

The Society then adjourned.

M. B. HUTCHINS, M. D.,
Secretary *pro tem.*

R. R. KIME, M. D., Reporter.

